# Relationship Between Patient-Provider Language Discordance and the Need for Professional Medical Interpretation for International Patients in Japan

**DOI:** 10.7759/cureus.47001

**Published:** 2023-10-13

**Authors:** Akira Oonishi, Ai Ikeda, Niyonsaba Francois, Naoko Ono

**Affiliations:** 1 Department of Medical Interpreting, Graduate School of Medicine, Juntendo University, Tokyo, JPN; 2 Astellas Pharma Inc., Medical Affairs Japan, Tokyo, JPN; 3 International Liberal Arts, Juntendo University, Tokyo, JPN

**Keywords:** medical interpreters, patient comprehension, language barrier, language discordance, language concordance

## Abstract

Background: Language barriers between patients and healthcare providers pose significant challenges in medical care in Japanese hospitals. Inadequate patient understanding of discussions with healthcare providers because of patient-provider language discordance has been reported in previous studies. There are growing expectations of professional medical interpreters to address these challenges. A previous study reported that patients with patient-provider language discordance were more likely to need interpreter assistance compared with patients with patient-provider language concordance.

Objective: We conducted a cross-sectional study utilizing a questionnaire survey of foreign patients to analyze the impact of the degree of patient-provider language discordance on the degree of patient comprehension of patient-provider communication, as well as patients’ need for professional medical interpreters in Japanese hospitals.

Method: From February 2022 to May 2023, an online questionnaire was distributed to 4,962 individuals aged 18 years or older who were non-native speakers of Japanese and who had attended medical institutions in Japan because of illness or injury experienced by themselves or by their children. A chi-square test and residual analysis were used to analyze the relationship between patient-provider language discordance and patient comprehension of patient-provider language concordance. Logistic regression analysis was performed to analyze the relationship between patient-provider language discordance and the necessity of professional medical interpretation.

Results: Among 4,962 study subjects who received the online survey, the total number of responses was 363 (7.3%). The rate of low-level patient comprehension was significantly higher in the patient-provider language discordance group compared with other groups. In a logistic regression model that accounted for sociodemographic factors, both the partial and complete patient-provider language discordance groups were more likely to want to use professional medical interpreters compared with the patient-provider language concordance group (OR: 4.16; 95% CI, 1.55-11.16; P=0.005; OR: 4.73; 95% CI, 1.70-13.18; P=0.003, respectively).

Conclusion: The current findings suggest that hospitals should be better prepared to meet the potential language needs of international patients with no or limited use of Japanese in daily conversation.

## Introduction

The number of foreign residents in Japan increased until the end of 2019 when it reached an all-time high of approximately 2.93 million foreign residents [1]. This increase was caused by deliberate measures by the Japanese government, including a plan to bring 300,000 international students and the creation of the Technical Intern Training Program and the Specified Skilled Worker System, as well as spontaneous growth resulting from globalization, the internationalization of Japanese companies, information technology workers, and international marriages [2]. Despite the coronavirus disease 2019 pandemic causing a decrease in the foreign resident population, which was 2.88 million at the end of 2020 and 2.76 million at the end of 2021, this population reached a record high of 3.08 million by the end of 2022 and is expected to continue to rise in the coming years [3,4]. With this increase in the foreign resident population, it is expected that the number of foreign patients seeking treatment at Japanese medical facilities will rise.

However, language barriers between patients and healthcare providers in Japan have been identified as a significant challenge. Inadequate patient understanding of discussions with healthcare providers because of patient-provider language discordance has been reported globally in previous studies, including surveys investigating healthcare for foreigners. Crane et al. conducted an interview survey with patients who received treatment from English-speaking healthcare providers in the emergency room at the time of discharge and reported that Spanish speakers had significantly lower correct response rates in understanding the treatment compared with English speakers [5]. Kazzi et al. indicated that guardians with limited English proficiency who brought children under the age of 14 to the emergency room had significantly poorer comprehension of the medical visit compared with guardians who were proficient in English, on the basis of interview surveys [6]. Lasater et al. found that Spanish speakers had a significantly poorer understanding of prescriptions than English speakers in a retrospective analysis of records of Hispanic patients aged 35-70 years old with type 2 diabetes using the United States public health system [7]. Dunlap et al. reported that, for families of Hispanic patients in a pediatric surgery clinic, Spanish speakers using interpreter services with an English-speaking medical team had a significantly less adequate understanding of medical information received during the visit compared with Spanish speakers using interpreter services with an English-speaking medical team [8].

Various types of interpretation are used as interventions to overcome language barriers. These encompass various methods, such as ad hoc interpretation provided by family or friends of the patient, the use of automatic translation devices like VoiceTra and POCKETALK, and the involvement of medical interpreters who possess professional training to enable effective communication through interpretation skills and medical knowledge [9].

Ad hoc interpretation is a relatively inexpensive and convenient method of interpretation for patients. However, several challenges have been reported, both domestically and internationally. Flores et al. reported that ad hoc interpreters have a significantly higher frequency of interpretation errors than trained interpreters, which can have a negative impact on clinical outcomes [10]. A survey of Brazilians by Nagata et al. found that ad hoc interpreters did not accurately convey the patient’s and physician’s words and lacked sufficient knowledge of relevant terminology [11].

While interpretation with automated translation devices can provide support for a wide range of languages, Patil et al. reported problems with the accuracy of these systems, including obvious mistranslations [12]. Nomura et al. reported that, while automatic translation devices may be sufficient for some medical treatment-related procedures (comprehensive guidance, reception, scheduling tests, and follow-ups), they are not sufficient for “disclosure of cancer or other serious diseases” [13].

Expectations are growing for medical interpreters, whose interpretation skills and medical knowledge bridge the communication gap between patients and providers, and whose usefulness has been reported internationally. Lindoholm et al. and Abbato found that non-English-speaking patients who used medical interpreters had significantly shorter hospital stays [14,15].

The need for medical interpreters under patient-provider language discordance has also been reported in previous research. In the study mentioned above, Kazzi et al. observed that patients with limited English proficiency were more likely to need interpreter assistance compared with patients with English proficiency (OR: 44.2; 95% CI, 21.6-90.7) [6]. A survey of hospitals by Hamai et al. also found that hospitals that took in patients for emergency room visits, hospitalization, testing, and surgery were more likely to see the need for professional medical interpreters, with 94% of hospitals with dispatch or in-house medical interpreters reporting that these services were needed [16].

Given this situation, we hypothesized that patient-provider language discordance may influence patients’ understanding of discussions between patients and healthcare providers, as well as the need for medical interpretation. However, to the best of our knowledge, no previous studies have investigated patient comprehension of patient-provider communication and the necessity of medical interpreters on the basis of the degree of patient-provider discordance.

Therefore, we conducted a cross-sectional study utilizing a questionnaire survey of foreign patients to analyze the impact of the degree of patient-provider language discordance on patient comprehension of patient-provider communication, as well as patients’ need for professional medical interpreters in Japanese hospitals.

## Materials and methods

Research subjects and survey methods

The study subjects were defined as individuals 18 years of age or older who visited a hospital in Japan for their own or their child’s illness or injury, and whose native language is not Japanese. Organizations such as international exchange associations and Japanese language support classes were identified, then requests for survey participation were made via email, and opportunities to explain the research were created through Zoom or face-to-face meetings with organizations that granted consent. After consent for the survey was obtained, a link to the online survey form (Google or WJX.CN.) was sent to the organizations. The online survey was made accessible through a QR code on the website created for this survey and on the physical flyer. The online survey was sent to 4,962 study subjects who were members of international organizations or Japanese language support classes (46 organizations), from February 2022 to May 2023.

Survey items

The survey in Google Forms asked about the experience of the subjects or their children when visiting hospitals in Japan and was created in four languages, Easy Japanese, English, Chinese, and Vietnamese, from which subjects were able to choose when responding. A question to confirm whether it is the first time to respond to this survey was set at the beginning of the online survey form so that the same person does not answer the questionnaire survey. The estimated time for completion was approximately 20 minutes. Survey items consisted of personal attributes (e.g., language used in daily life, country of origin, age, length of residency, sex), details of their visit (e.g., hospital location, degree of patient comprehension of doctor-patient communication on a four-item scale), Illness perception scale, patient satisfaction questionnaire, and understanding of and interest in medical interpretation services and communication with physicians [17,18].

Patient-provider concordance group was determined using the question “What language do you usually use in your daily life?” with the following answer options: (a) Japanese, (b) English, (c) Chinese, (d) Vietnamese, and (e) other. Although these questions allowed multiple answers, in this study we classified the selection of (a) only as “patient-provider language concordance group,” responses that included (a) as “partial patient-provider language discordance group” and other responses as “patient-provider language discordance group.”

The degree of patient comprehension of patient-provider communication was determined with the question “How well did you understand the content of the conversation with the healthcare professional (doctor)?” on a scale from level one (did not understand at all) to level four (completely understand). Individuals with level one or two conversational comprehension were classified as the low-level patient comprehension group, while those with level three or four comprehension were classified as the high-level patient comprehension group.

The need for professional medical interpreters was determined with the question “When using a Japanese hospital, would you like to use a medical interpreter service?” with the following answer options: (a) I would like to use this service, (b) I would like to use this service in the language I use on a daily basis if it is available, (c) I would like to use this service if it is cheap (or free of charge), and (d) the service is unnecessary. Although these questions allowed multiple answers, in this study we classified the selection of (d) only as no and all other answers as yes. The items of the illness perception scale (B-IPQ) and patient satisfaction questionnaire were not covered in this study.

Statistical analysis methods

In the cross-tabulation of basic attributes and the degree of language discordance, differences in basic attributes (e.g., age, sex, country of origin, age, duration of stay, residency status) on the basis of patient-provider language concordance, partial patient-provider language discordance, and patient-provider language discordance group were analyzed. The Mann-Whitney U test was used for age, whereas the chi-squared test was used for other variables.

In the cross-tabulation of the degree of patient-provider language discordance and patient comprehension of patient-provider communication, a chi-squared test and residual analysis were used to analyze the relationship between the degree of patient-provider language discordance and the degree of patient comprehension of patient-provider communication.

Logistic regression analysis was conducted with the need for a medical interpreter as the objective variable, and the covariates (explanatory variables) were sex, age, country of birth, and the degree of patient-provider language discordance. IBM SPSS STANDARD 29.0 was employed for the chi-squared test, Mann-Whitney U test, and logistic regression analysis, with a statistical significance level of 5%.

Ethical considerations

As part of the questionnaire’s specifications, “Agree” and “Disagree” options were provided before starting the questionnaire on Google Forms, and only those who selected “Agree” were allowed to proceed to the actual questions, thereby ensuring that each subject had the opportunity to personally decide whether to participate in this study. The implementation plan for this study was approved by the Research Ethics Committee of the Faculty of Medicine at Juntendo University (Approval: January 19, 2023 [“Ver. 3”], Approval No: E21-0237).

## Results

Among 4,962 individuals who received an online questionnaire survey, a total of 363 responses were collected, with a response rate of 7.3%. The rest of the 4,599 individuals did not respond to this questionnaire survey for any reason based on their free decision, although the specific reasons were not identified through this questionnaire survey. The number of valid responses, excluding 17 samples that did not meet the research subject selection criteria, five samples in which gender was not specified, and four samples with missing data for the question regarding the need for professional medical interpreters, was 337 (92.8% of the total responses).

Sociodemographic characteristics of international patients

Table 1 shows a collation of patients’ sociodemographic data, including self-reported age, sex, country of origin, hospital area, duration of stay, and recipient of medical care stratified into a patient-provider language concordance group, a partial patient-provider language discordance group, and a patient-provider language discordance group. There were significant sociodemographic differences in patient-provider language concordance, partial patient-provider language discordance, and patient-provider language discordance group with regards to age (P<0.001), country of birth (P<0.001), hospital area (P=0.03), and use of interpretation (P=0.003).

**Table 1:** Background factors by the degree of patient-provider language concordance discordance group *The Mann-Whitney U test was used for examining age, while the chi-squared test was used for examining the other variables

	Overall (n=337)	Patient-provider language concordance (n=23)	Partial patient-provider language discordance (n=181)	Patient-provider language discordance (n=133)	P-value*
Age, y, mean (SD)	31.38 (11.03)	37.00 (13.01)	28.96 (9.70)	33.70 (11.60)	<0.001
Sex, n (%)					0.80
Male	149 (44.2)	11 (47.8)	82 (45.3)	56 (42.1)	
Female	188 (55.8)	12 (52.2)	99 (54.7)	77 (57.9)	
Country of birth, n (%)					< 0.001
China	124 (36.8)	7 (30.4)	80 (44.2)	37 (27.8)	
Vietnam	127 (37.7)	6 (26.1)	73 (40.3)	48 (36.1)	
Other	86 (25.5)	10 (43.5)	28 (15.5)	48 (36.1)	
Hospital area, n (%)					0.03
Tokyo	168 (49.9)	13 (56.5)	89 (49.2)	66 (49.6)	
Chiba, Kanagawa, Saitama	43 (12.8)	7 (30.4)	23 (12.7)	13 (9.8)	
Other	126 (37.4)	3 (13.0)	69 (38.1)	54 (40.6)	
Duration of stay, n (%)					0.71
Less than one year	43 (12.8)	2 (8.7)	22 (12.2)	19 (14.3)	
More than one year	294 (87.2)	21 (91.3)	159 (87.8)	114 (85.7)	
Patient, n (%)					0.60
Respondent	312 (92.6)	21 (91.3)	170 (93.9)	121 (91.0)	
Respondent’s child	25 (7.4)	2 (8.7)	12 (9.0)	12 (9.0)	
Use of interpretation					0.003
No interpretation	220 (65.3)	19 (82.6)	128 (70.7)	73 (54.9)	
Any interpretation	117 (34.7)	4 (17.4)	53 (29.3)	60 (45.1)	

Relationship between the degree of patient-provider language discordance and patient comprehension of patient-provider communication

47.4% of the patient-provider discordance group was in the low-level patient comprehension group, while 26.1% of the patient-provider concordance group was in the low-level patient comprehension group (Table 2). We reviewed the relationship between the degree of patient-provider language discordance and patient comprehension of patient-provider communication using the chi-squared test and residual analysis in cross-classified tables. We found that the rate of low-level patient comprehension was significantly higher in the patient-provider language discordance group compared with other groups (2.3 of residual analysis, Table 2), and the rate of high-level patient comprehension was significantly lower in the patient-provider language discordance group compared with other groups (−2.3 of residual analysis, Table 2).

**Table 2:** Patient comprehension of patient-provider communication by the degree of patient-provider language discordance *Variables considered statistically significant if adjusted residuals are ≥1.96 or ≤−1.96 ^†^Chi-square test was used

	Low-level patient comprehension group (n = 134)			High-level patient comprehension group (n = 203)			Total (n = 337)		P Value^†^
	n	%	Adjusted residual*	n	%	Adjusted residual*	n	%	0.047
Patient-provider language concordance	6	26.1	−1.4	17	73.9	1.4	23	100.0	
Partial patient-provider language discordance	65	35.9	−1.6	116	64.1	1.6	181	100.0	
Patient-provider language discordance	63	47.4	2.3	70	52.6	−2.3	133	100.0	

Relationship between the degree of patient-provider language discordance and the need for professional medical interpreters

In a logistic regression model that accounted for sociodemographic factors such as age, sex, country of birth, and the degree of patient-provider language discordance, both the partial and complete patient-provider language discordance groups were more likely to want to utilize professional medical interpreters compared with the patient-provider language concordance group (OR: 4.16; 95% CI, 1.55-11.16; P=0.005; OR: 4.73; 95% CI, 1.70-13.18; P=0.003, respectively; see Table 3). Similarly, there was a significant relationship between sex or country of birth and patients’ need for the use of professional medical interpreters in Japanese hospitals.

**Table 3:** Association of the degree of patient-provider language discordance and need for professional medical interpreters (logistic regression analysis)

Variables	Classification	OR	95% CI	P-value
Degree of patient-provider language discordance	Concordance	ref		
	Partial discordance	4.16	1.56-11.16	0.005
	Discordance	4.73	1.70-13.18	0.003
Age		0.98	0.95-1.01	0.29
Sex	Male	ref		
	Female	0.50	0.26-0.93	0.03
Country of birth	China	ref		
	Vietnam	0.35	0.13-0.83	0.03
	Others	0.77	0.33-1.82	0.55
Hospital area	Tokyo	ref		
	Chiba, Kanagawa, Saitama	1.44	0.53-3.87	0.47
	Others	1.07	0.43-2.65	0.88
Use of interpretation	No interpretation	ref		
	Any interpretation	1.56	0.80-3.06	0.20

## Discussion

Summary of findings

This study aimed to analyze the impact of the degree of patient-provider language discordance on the degree of patient comprehension of patient-provider communication as well as patients’ need for the use of professional medical interpreters. Although the rate of interpretation provided for the patient-provider language discordance group was higher compared with other groups, the rate of low-level patient comprehension was significantly higher in the patient-provider language discordance group compared with other groups. Crane et al. reported that the average percentage of correct responses to the questions about diagnosis, medication, and medication function to Spanish patients discharged from the Emergency Department in Kern Medical Center in California was significantly lower than that of correct responses to the questions to English patients (46% vs. 65%, P<0.001), which is consistent with our study results [5]. Kazzi et al. reported that respondents in Australia with limited English proficiency were more likely to have an impaired understanding of the consultation compared with respondents with English proficiency (OR 8.2; 95% CI 4.7-14.1) [6]. Lasater et al. reported that Spanish-speaking patients in the United States were less likely to understand their prescriptions compared with English-speaking patients (22% of Spanish-speaking patients with no comprehension vs. 3% of English-speaking patients with no comprehension, P=0.001) [7].

In a logistic regression analysis in our study, the partial patient-provider language discordance group and the patient-provider language discordance group were more likely to want to utilize professional medical interpreters, compared with the patient-provider language concordance group. Kazzi et al. also reported that patients with limited English proficiency were more likely to need interpreter assistance compared with patients with English proficiency (OR, 44.2; 95% CI 21.6-90.7), in accordance with the current results [6]. In a study in Japan, Hamai et al. reported that when foreign patients were admitted to hospitals, 75.7%, 84.7, and 94.7% of small, medium, and large hospitals required trained medical interpreters, respectively [16]. To the best of our knowledge, however, the current study is the first demonstration of the relationship between the degree of patient-provider language discordance and the need for professional medical interpretation in foreign patients in Japan.

Clinical implications

The current results revealed that patients with patient-provider language discordance who do not use Japanese in everyday conversation at all experienced significantly lower comprehension of discussions with healthcare providers. Additionally, the results indicated that both complete or partial patient-provider language discordance with no or limited use of Japanese in daily conversation contributed to the need for professional medical interpreters.

While demand for medical interpreters is high, in actual clinical practice, medical interpreters are underutilized, even when patient-provider language discordance is recognized. Among the 337 participants in this study, only seven (2.1%) utilized professional medical interpreters. The Japanese Ministry of Health, Labour, and Welfare also reported that the utilization rate of professional medical interpreters among 1,710 medical institutions across Japan was 12.7% [19]. In the study by Kazzi et al. mentioned earlier, only 47 (35.9%) out of 131 respondents required interpretation and utilized professional medical interpreters [6].

These findings suggest that medical institutions in Japan should be prepared to provide medical interpreters for patients who do not use Japanese in everyday conversation at all or use it only partially and should offer the option of using medical interpreters as needed to patients.

Limitations

This study involved several limitations. First, the number of participants was relatively small, especially for the patient-provider language concordance group. Thus, the analysis may have been underpowered for detecting a clinically meaningful relationship between the degree of patient-provider language discordance and patient comprehension of patient-provider communication or the need for professional medical interpreters. Second, this cross-sectional study was only able to examine comparisons and associations and not causality between patient-provider language discordance and patient comprehension of patient-provider conversation or the need for professional medical interpretation. Third, these and other unmeasured confounders may limit the ability to generalize our findings to medical institutions in Japan and other countries. We propose that future large-sample studies should aim to systematically understand linguistic needs and patient comprehension of doctor’s consultations depending on the degree of patient-provider language discordance. However, this study is the first of its kind in Japan to indicate that the degree of patient-provider language discordance may influence patient comprehension of patient-provider communication or the need for professional medical interpreters for international patients from a patient perspective.

## Conclusions

In the current study, we found that international patients with no use of Japanese in daily conversation were less likely to comprehend patient-provider conversations, and those with no or limited use of Japanese were more likely to need professional medical interpreters. These findings highlight the need to be better prepared to meet patients’ language needs for patients with no or limited use of Japanese in daily conversation. Further research will be required to systematically understand patient comprehension of patient-provider comprehension and linguistic needs on the basis of the degree of patient-provider language discordance.
